# Piroxicam fails to reduce myocellular enzyme leakage and delayed onset muscle soreness induced by isokinetic eccentric exercise

**DOI:** 10.1155/S0962935196000336

**Published:** 1996-06

**Authors:** J-L. Croisier, G. Camus, T. Monfils, G. Deby-Dupon, M. Fafchamps, I. Venneman, J-M. Crielaard, A. Juchmès-Ferir, C. Lhermerout, M. Lamy, C. Deby

**Affiliations:** 1 Department of Physical Medicine and Rehabilitation Institute of Chemistry Belgium; 2 Center for the Biochemistry of Oxygen Institute of Chemistry Belgium; 3 Department of Anesthesiology and Intensive Care Medicine ISEPK Belgium; 4 Laboratory of Human Applied Physiology ISEPK Belgium; 5 Department of Clinical Biology CHU University of Liège Sart Tilman Liège B-4000 Belgium

## Abstract

To test the hypothesis that delayed onset muscular soreness (DOMS) following intense eccentric muscle contraction could be due to increased production of prostaglandin E_2_ (PGE_2_), ten healthy male subjects were studied. Using a double-blind randomized crossover design, each subject performed two isokinetic tests separated by a period of at least 6 weeks: once with placebo, and once with piroxicam (Feldene^®^). They were given one capsule containing either placebo or piroxicam (20 mg) per day for 6 days with initial doses given starting 3 days prior to isokinetic testing. Exercise consisted of eight stages of five maximal contractions of the knee extensor and flexor muscle groups of both legs separated by 1 min rest phases, on a Kin Trex device at 60^°^/s angular velocity. The subjective presence and intensity of DOMS were evaluated using a visual analogue scale immediately after, and 24 and 48 h after each test. The mean plasma concentration of PGE_2_ measured at rest and after exercise was significantly lower in the group treated with piroxicam (p < 0.05). However, statistical analysis (two-way *ANOVA* test) revealed that exercise did not cause any significant change of mean plasma PGE_2_ over time in either of the two groups. Eccentric work was followed by severe muscle pain in extensor and flexor muscle groups. Maximal soreness was noted 48 h postexercise. Serum creatine kinase activity and the serum concentration of myoglobin increased significantly, and reached peak values 48 h after exercise in both experimental conditions (p < 0.001). By paired *t*-test, it appeared that there were no significant differences in the serum levels of these two markers of muscle damage between the two groups at any time point. We conclude that: (1) oral administration of piroxicam fails to reduce muscle damage and DOMS caused by strenuous eccentric exercise; and (2) the hypothetical role of increased PGE_2_ production in eccentric exercise-induced muscle damage, DOMS, and reduced isokinetic performance is not substantiated by the present results.


Mediators of Inflammation, 5, 230–234 (1996)

### Piroxicam fails to reduce myocellular enzyme leakage and delayed onset muscle soreness induced by isokinetic eccentric exercise

J-L. Croisier,[1] G. Camus,[2,4,CA] T. Monfils,[1,4]

G. Deby-Dupon,[2,3] M. Fafchamps, I. Venneman,[3]

J-M. Crielaard,[1] A. Juchmès-Ferir,[5]

C. Lhermerout,[4] M. Lamy[2,3] and C. Deby[2]

[1]Department of Physical Medicine and Rehabilitation, [2]Center for the Biochemistry of Oxygen, Institute of Chemistry, [3]Department of Anesthesiology and Intensive Care Medicine, [4]Laboratory of Human Applied Physiology, ISEPK and [5]Department of Clinical Biology, CHU, University of Liège, Sart Tilman, B-4000 Liège, Belgium

[CA]Corresponding Author

**To test the hypothesis that delayed onset muscular soreness (DOMS) following intense eccentric muscle contraction could be due to increased production of prostaglandin $E_2$ ($PGE_2$), ten healthy male subjects were studied. Using a double-blind randomized crossover design, each subject performed two isokinetic tests separated by a period of at least 6 weeks: once with placebo, and once with piroxicam (Feldene®). They were given one capsule containing either placebo or piroxicam (20 mg) per day for 6 days with initial doses given starting 3 days prior to isokinetic testing. Exercise consisted of eight stages of five maximal contractions of the knee extensor and flexor muscle groups of both legs separated by 1 min rest phases, on a Kin Trex device at 60°/s angular velocity. The subjective presence and intensity of DOMS were evaluated using a visual analogue scale immediately after, and 24 and 48 h after each test. The mean plasma concentration of $PGE_2$ measured at rest and after exercise was significantly lower in the group treated with piroxicam ($p < 0.05$). However, statistical analysis (two-way *ANOVA* test) revealed that exercise did not cause any significant change of mean plasma $PGE_2$ over time in either of the two groups. Eccentric work was followed by severe muscle pain in extensor and flexor muscle groups. Maximal soreness was noted 48 h post-exercise. Serum creatine kinase activity and the serum concentration of myoglobin increased significantly, and reached peak values 48 h after exercise in both experimental conditions ($p < 0.001$). By paired *t*-test, it appeared that there were no significant differences in the serum levels of these two markers of muscle damage between the two groups at any time point. We conclude that: (1) oral administration of piroxicam fails to reduce muscle damage and DOMS caused by strenuous eccentric exercise; and (2) the hypothetical role of increased $PGE_2$ production in eccentric exercise-induced muscle damage, DOMS, and reduced isokinetic performance is not substantiated by the present results.**

**Key words**: Delayed onset muscle soreness, Eccentric contraction, Isokinetic exercise, Muscle damage, Non-steroidal anti-inflammatory agent, Prostaglandin

### Introduction

Delayed onset muscle soreness (DOMS) refers to a dull, aching pain, frequently combined with tenderness and stiffness, localized to groups of previously active muscles. These symptoms are usually observed following strenuous, unaccustomed exercise, especially when eccentric contractions are involved.[1,2] DOMS sensations develop in the first 24 h following muscular work and subside within 5 to 7 days after exercise.[3]

Despite the volume of data that have accumulated concerning DOMS during the last decade, the underlying mechanisms of this phenomenon remain poorly understood. Based on previous studies showing that strenuous exercise causes muscle and connective tissue damage, as well as an inflammatory response involving the release of algogenic inflammatory mediators such as prostaglandins (PG),[4–7] it has been hypothesized that increased PG production by activated macrophages in injured areas could be one of the main

© 1996 Rapid Science Publishers chemical stimuli responsible for DOMS.[8] Histological and biochemical indices of exercise-induced muscle damage,[9–16] the presence of leucocytes capable of producing arachidonic acid (AA) derived products in damaged muscles,[14,15,17] and the increased production of these substances during exercise[4–7] support this hypothesis. The results of a recent human study, where ibuprofen, a potent nonsteroidal anti-inflammatory drug (NSAID), reduced both DOMS and the decrease of muscle performance after strenuous eccentric contractions[18] argued also for the involvement of PGs. It should be pointed out, however, that the effects of inhibitors of AA metabolism on DOMS is a matter of controversy. Several research groups have reported that anti-inflammatory agents inhibiting the production of AA-derived compounds failed to alleviate soreness after exercise.[13,19,20] Among the possible factors responsible for these discrepancies are the administration protocol of NSAIDs and the effective doses that are absorbed. Therefore, in an attempt to address the question of whether metabolites of AA derived from the cyclooxygenase pathway, especially $PGE_2$, are involved in DOMS, we studied the effects of piroxicam (Feldene®), another potent NSAID, on perceived soreness, biochemical indices of muscle damage, and plasma levels of $PGE_2$ in subjects submitted to maximal eccentric isokinetic contractions. To optimize the potential effects of piroxicam on these variables, the drug was administered both before (prophylactically) and after (therapeutically) to each subject.

### Methods

*Subjects:* Ten moderately active, healthy male subjects volunteered to take part in this study after giving informed consent. Their mean age (± S.E.M.) and body mass were 22 ± 0.4 years and 74 ± 1.5 kg, respectively. None was currently involved in lower body resistance or endurance training. They were requested to refrain from strenuous exercise and to abstain from the consumption of any form of medication during the study period. The experimental protocol was approved by the Ethics Committee of the University Hospital Center of Liège.

*Experimental procedures:* All isokinetic measurements were performed on a Kin Trex device (Meditronic Instruments SA, Ecublens, Switzerland) at 60°/s angular velocity. During these experiments, the subjects performed isokinetic exercise in a seated position at approximately 105° of hip flexion. Belts placed across hips, chest and thigh were used to stabilize their position.

On their first visit to the laboratory, the subjects were familiarized with the experimental procedures and possible risks involved. They were accustomed to the isokinetic device by performing 10 to 20 submaximal eccentric contractions of quadriceps and hamstring muscles. On their second visit, the eccentric and concentric maximum peak torque (PTmax) of the knee flexor and extensor muscle groups were measured bilaterally as follows. Standard warm-up exercises consisted of 5 min cycling at 75–100 W on a bicycle ergometer (60 rpm) and stretching of the muscle groups subsequently used for isokinetic contractions. This warm-up protocol was systematically applied before each isokinetic trial. After a 5 min recovery phase, the subjects performed three maximal contractions of knee flexor and extensor muscles at 60°/s in the eccentric mode. The greatest peak torque values were taken to represent PTmax. At least 2 weeks after this first test, the volunteers were randomly divided into two groups of five subjects who were given six capsules containing either 20 mg of piroxicam or a placebo. Using a double-blind procedure in which neither the subjects nor the investigator providing the capsules were aware of the subjects assigned group, the volunteers were instructed to take one capsule per day, starting 3 days before the isokinetic sessions. Each subject served as his own control, and completed two study protocols separated by at least 6 weeks: once with placebo and once with piroxicam. Exercise consisted of eight stages of five maximal contractions of the knee extensor and flexor muscle groups of both legs, separated by 1 min rest phases. To assess the effects of isokinetic contractions on muscle function, PTmax was measured on day two after exercise testing, using the protocol described above.

*Perception of muscle soreness and indirect evaluation of muscle damage:* The subjective presence and intensity of DOMS were evaluated using a visual analogue scale graded from 0 (no pain) to 10 (very severe, intolerable pain) immediately after, and 24 and 48 h after eccentric testing. Increased serum levels of creatine kinase (CK) and myoglobin (Mb) were used as indirect indices of exercise-induced muscle damage.

*Blood sampling:* Before each test, an indwelling catheter was inserted into an antecubital vein and sealed. Venous blood samples (10 ml) were collected into sterile tubes immediately before isokinetic test, 10 min after catheter insertion; immediately after exercise, and after 30 min recovery. Two additional blood samples were taken by venipuncture 24 and 48 h after exercise, respectively. The venous blood samples were divided in two aliquots: 5 ml whole fresh blood into a plain plastic tube, and 5 ml of whole blood into a vial containing EDTA as anti-coagulant. The first portion was allowed to clot at room temperature, and the serum phase was used for the measurement of creatine kinase (CK) activity and myoglobin (Mb) concentration. The second portion was immediately centrifuged. The supernatants were kept at –70°C until plasma $PGE_2$ assay.

*Biochemical assays:* Plasma $PGE_2$ levels were assessed using an enzyme immunoassay (Cayman Chemical, Kit 514016). Routine spectrophotometric techniques used in the Dept of Clinical Biology were applied to measure serum CK activity and Mb concentrations.

*Statistical analysis:* Values in the text and figures are expressed as group means ± S.E.M. For each variable, differences between mean values obtained at each time point in the two groups were evaluated using a paired Student's *t*-test. For the variables listed above, a two-way analysis of variance for repeated measures design (two-way ANOVA test) was used to assess changes over time. The level of statistical significance was set to $p < 0.05$.

### Results

Mean peak torque produced by the quadriceps and hamstring muscle groups during pre- and post-test exercise sessions in the two experimental conditions are shown in Fig. 1. Pre-test mean PTmax of knee flexor and extensor muscle groups were 157 ± 9 and 339 ± 17 Nm, respectively. Maximum eccentric contractions were not followed by any significant change of this variable after two days of recovery.

As shown in Fig. 2, eccentric exercise caused marked soreness, reaching peak levels 2 days after isokinetic testing. The pattern of change of mean soreness scores over time, as well as mean soreness scores at each time point were not significantly changed by piroxicam. The effects of this agent on mean plasma $PGE_2$ concentrations are illustrated in Fig. 3. When compared to the placebo group, this variable was significantly decreased at four time points in the piroxicam group ($p < 0.05$). By the two-way ANOVA test, it appeared that exercise did not cause any significant change of plasma $PGE_2$ concentration over time in either experimental condition.

The effects of exercise on serum levels of CK and Mb are shown in Fig. 4 and 5, respectively. Beginning on the day after the isokinetic test, marked release of CK was observed in both groups, reaching a maximum on day 2 of recovery ($p < 0.001$). Serum Mb concentration also demonstrated a delayed peak after 48 h recovery in both experimental conditions ($p < 0.001$). Piroxicam did not significantly change the mean serum levels of these biochemical markers of muscle damage at any time point.

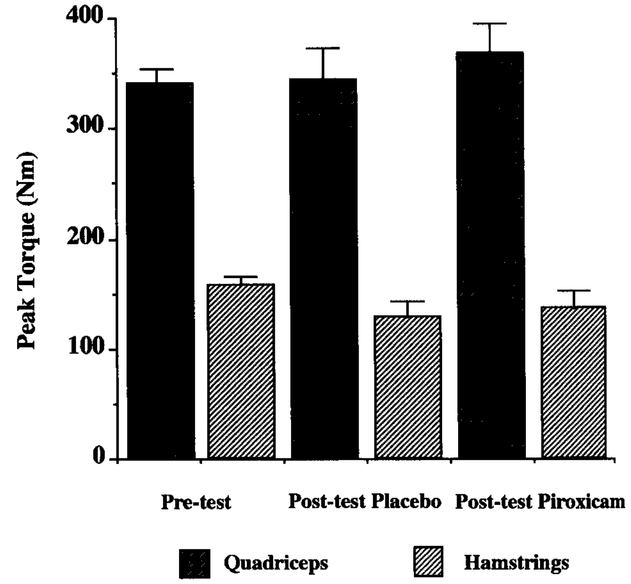

FIG. 1. Peak torque produced by quadriceps and hamstring muscle groups in the eccentric mode before and 2 days after maximal eccentric tests in the two experimental conditions.

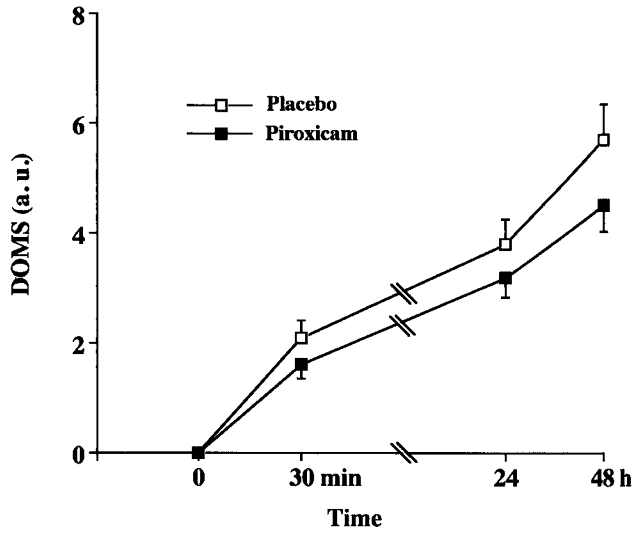

FIG. 2. Mean soreness rating score (arbitrary units) of the quadriceps and hamstring muscle groups before and after eccentric exercise bouts in the two experimental conditions. 0: Pre-exercise; 30 min: immediately after exercise; 24 h, 48 h: 24 h and 48 h after exercise.

### Discussion

The marked increase of serum CK activity and Mb concentration following isokinetic eccentric

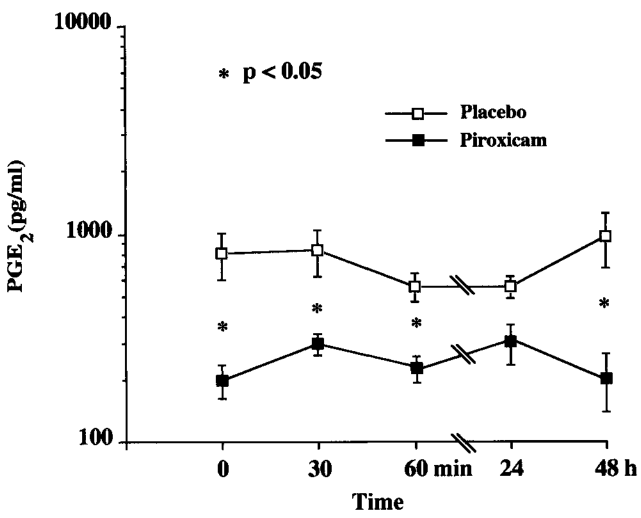

FIG. 3. Plasma concentration of prostaglandin $E_2$ as a function of time in the two experiments. Vertical bars are S.E.M. 0: Pre-exercise; 30 min: immediately after exercise; 60 min: after 30 min recovery; 24 h, 48 h: 24 h and 48 h after exercise.

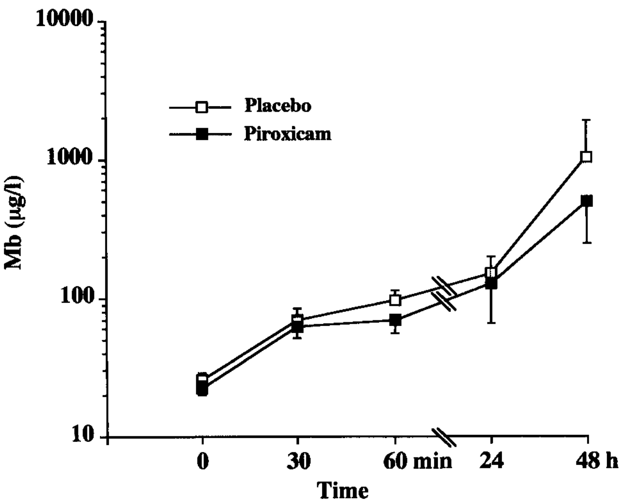

FIG. 5. Mean serum concentration of myoglobin (logarithmic scale) as a function of time in the placebo and piroxicam groups (see Fig. 3).

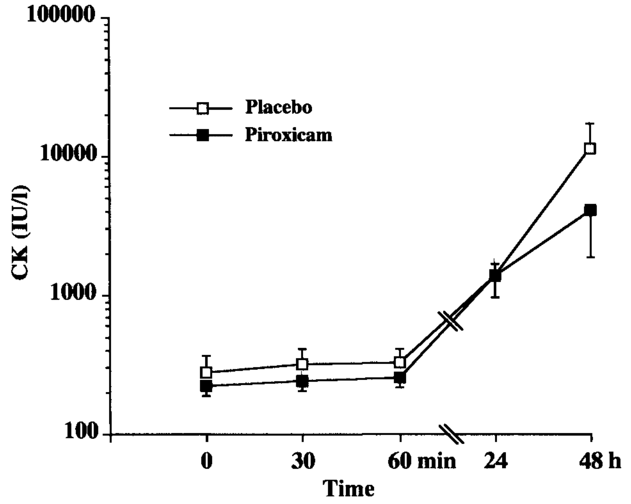

FIG. 4. Mean serum activity of creatine kinase (logarithmic scale) as a function of time in the placebo and piroxicam groups (see Fig. 3).

exercise is in agreement with the results of previous studies.[1,3,10,15,18,21] This protein leakage from skeletal muscle has been widely used to estimate the severity of exercise-induced skeletal muscle damage. In accordance with this view, the data presented in Figs 4 and 5 indicate that the present exercise protocol caused severe myocellular injury. Surprisingly, when compared to the PTmax measured before isokinetic exercise, the ability of the knee flexor and extensor muscle groups to produce force was unaffected 2 days after the damage induced by the two experimental conditions. This suggests that muscle injury was not severe enough to alter PTmax and/or that motor units containing non-damage fibres were recruited.

Despite the large number of studies currently available concerning DOMS, little is known about its underlying mechanisms. Based on literature data showing that eccentric muscular work is the most effective way to cause muscle damage associated with humoral and cellular indices of an inflammatory response, it has been suggested that algogenic substances derived from arachidonic acid metabolism, especially $PGE_2$, could play a pivotal role in the production of the delayed sensation of pain,[8] a cardinal symptom of DOMS. The increase of muscle AA levels accompanying static contraction,[22] the production of prostaglandins during strenuous muscular work,[4–7] as well as reports of the beneficial effects of NSAIDs on exercise-induced injuries in mouse skeletal muscle fibres[23] and certain manifestations of DOMS in man,[18,21,24] lend support to this hypothesis. However, previous studies suggesting that other factors could also be involved in exercise-induced pain, as well as the discrepancies concerning the effects of drugs known to inhibit the AA metabolism on DOMS led us to verify whether piroxicam could effectively help to manage pain, reduced muscle performance and muscle damage following maximal isokinetic contractions in the eccentric mode. To optimize the inhibitory action of this agent on $PGE_2$ synthesis, six doses of 20 mg piroxicams daily, with initial dose given 3 days before the exercise tests were administered. The significant reduction of plasma $PGE_2$ concentrations demonstrates the efficacy of cyclooxygenase blockade. However, piroxicam administered had no effect on muscle enzyme leakage and DOMS following isokinetic eccentric contractions. These findings lead us to conclude that $PGE_2$ (and probably other compounds) derived from AA

metabolism via the cyclooxygenase pathway do not play a major role in the pathogenesis of DOMS nor in exercise-induced damage. Nevertheless, the hypothesis that larger doses of piroxicam or lowering the plasma levels of $PGE_2$ below 200 pg/ml could reduce DOMS in subjects submitted to damaging exercise of longer duration deserves to be tested experimentally.

In contrast with previous studies where strenuous exercise was found to increase plasma and muscle levels of AA-derived prodcuts,[4–7] there was no significant change in plasma $PGE_2$ concentration over time in the two experimental conditions. The reasons for this discrepancy are unclear. One possibility is that we measured plasma levels of $PGE_2$ in venous blood samples drawn at a site distant from the active muscles in which this compound has been shown to accumulate.[7] Because of the short half-life of $PGE_2$ in blood, it is possible that regional changes of this variable in the venous effluent of skeletal muscles are not detectable in blood from a forearm vein. Another factor that could act in concert with the sampling site effects is the limited muscle mass involved in the isokinetic exercise. The isokinetic device used in the present study does not allow simultaneous contraction of limb muscles on both sides. It is therefore possible that the amount of $PGE_2$ released from the active muscles was too low to be reflected by changes in venous $PGE_2$ concentrations measured in blood samples drawn from a forearm vein. For ethical and technical reasons, we did not measure the arterio-venous difference of $PGE_2$ at the lower limbs. Therefore, increased production of this compound induced by exercise in the active muscles cannot be definitively ruled out. Nevertheless, the significant difference of resting and post-exercise plasma concentrations of $PGE_2$ between the two groups demonstrates that the dose of piroxicam used in this study effectively inhibited the production of this compound. As mentioned above, because the failure to find a significant difference in either the serum levels of the biochemical markers of muscle injury or in the intensity of DOMS between the two groups, despite piroxicam-induced reduction of plasma $PGE_2$ concentrations, argues against the hypothesis that cyclooxygenase-derived AA metabolism are involved in exercise-induced damage and DOMS. Based on these results, it would appear that the activation of pain afferents after eccentric contractions is caused by other noxious stimuli. It has been suggested that increased intramuscular pressure,[2] and/or production of algogenic substances such as serotonin, histamine, acetylcholine and kinins could be involved in the production of DOMS.[8] To our knowledge, these hypotheses remain to be tested experimentally.

### References

1. Armstrong RB. Initial events in exercise-induced muscular injury. *Med Sci Sports Exerc* 1990; **22**: 429–435.
2. Garrett WE, Califf JC, Bassett FH. Histochemical correlates of hamstring injuries. *Am J Sports Med* 1984; **12**: 98–103.
3. Ebbeling CB, Clarkson PM. Exercise-induced muscle damage and adaptation. *Sports Med* 1989; **7**: 207–234.
4. Demers LM, Harrison TS, Halbert DR, Santen RJ. Effect of prolonged exercise on plasma prostaglandin levels. *Prostaglandin Med* 1981; **6**: 413–418.
5. Hikida RS, Staron RS, Hagerman FC, Sherman WN, Costill DL. Muscle fibre necrosis associated with human marathon runners. *J Neurol Sci* 1983; **59**: 185–203.
6. Round JM, Jones DA, Cambridge G. Cellular infiltrates in human skeletal muscle: exercise induced damage as a model for inflammatory muscle disease? *J Neurol Sci* 1987; **82**: 1–11.
7. Young EW, Sparks HV. Prostaglandin E release from dog skeletal muscle during restricted flow exercise. *Am J Physiol* 1979; **236**: H596–H599.
8. Symons JD, Theodossy SJ, Longhurst JC, Stebbins CL. Intramuscular accumulation of prostaglandins during static contraction of the cat triceps surae. *J Appl Physiol* 1991; **71**: 1837–1842.
9. Camus G, Deby-Dupont G, Duchateau J, Deby C, Pincemail J, Lamy M. Are similar inflammatory factors involved in strenuous exercise and sepsis? *Intensive Care Med* 1994; **20**: 602–610.
10. Croisier J-L, Camus G, Deby-Dupont G, Bertrand F, Crielaard J-M, Lhermerout C, Lamy M. Muscle damage and delayed muscular soreness induced by eccentric isokinetic exercise. *Pflügers Archiv* 1995; **429**: R11.
11. Friden J, Skafianos PN, Hargens AR. Delayed muscle soreness and intramuscular fluid pressure: comparison between eccentric and concentric load. *J Appl Physiol* 1986; **61**: 2175–2179.
12. Hasson SM, Daniels JC, Divine JG, Niebuhr BR, Richmond S, Stein PG, Williams JH. Effect of ibuprofen use on muscle soreness, damage, and performance: a preliminary investigation. *Med Sci Sports Exerc* 1993; **25**: 9–17.
13. Herbaczynska-Cedro K, Staszewska-Barczak J, Janczewska H. Muscular work and the release of prostaglandin-like substances. *Cardiovasc Res* 1976; **10**: 413–420.
14. Jones DA, Newham DJ, Round JM, Tolfree SEJ. Experimental human muscle damage morphological changes in relation to other indices of damage. *J Physiol Lond* 1986; **375**: 435–448.
15. Kuipers H, Keizer HA, Verstappen FTJ, Costill DL. The influence of a prostaglandin inhibiting drug on muscle soreness after eccentric work. *Int J Sports Med* 1985; **6**: 336–339.
16. Nowak J, Wennmalm A. Effect of exercise on human arterial and regional venous plasma concentration of prostaglandin E. *Prostaglandin Med* 1978; **1**: 489–497.
17. Rotto DM, Massey KD, Burton KD, Kaufman MP. Static contraction increases arachidonic acid levels in gastrocnemius muscles of cats. *J Appl Physiol* 1989; **66**: 2721–2724.
18. Headley SA, Newham DJ, Jones DA. The effects prednisolone on exercise induced muscle pain and damage. *Clin Sci* 1986; **70**: 85P.
19. Camus G, Pincemail J, Deby-Dupont G, Deby C, Juchmès-Ferir A, Lamy M. Effects of methylprednisolone on exercise-induced increases of polymorphonuclear elastase and myeloperoxidase in man. Preliminary results. *Mediators of Inflammation* 1993; **2**: 323–326.
20. Newham DJ, Jones DA, Edwards RHT. Plasma creatine kinase changes after eccentric and concentric contractions. *Muscle Nerve* 1986; **9**: 59–63.
21. Donnelly AE, McCormick K, Maughan RJ, Whiting PH, Clarkson PM. Effects of non-steroidal anti-inflammatory drug on delayed onset muscle soreness and indices of damage. *Br J Sports Med* 1988; **22**: 35–38.
22. Salminen A, Kihlström M. Protective effect of indomethacin against exercise-induced injuries in mouse skeletal muscle fibres. *Int J Sports Med* 1987; **8**: 46–49.
23. Smith LL. Acute inflammation: the underlying mechanism in delayed onset muscle soreness? *Med Sci Sports Exerc* 1991; **23**: 542–551.
24. Francis KT, Hoobler T. Effects of aspirin on delayed muscle soreness. *J Sports Med* 1987; **27**: 333–337.
25. Friden J, Sjostrom M, Ekblom B. Myofibrillar damage after intense eccentric exercise in man. *Int J Sports Med* 1983; **4**: 170–176.

ACKNOWLEDGEMENTS. The authors wish to thank Yvette Goutman, Monique Dister and Josiane Bourdon-Neuray for expert technical assistance.

**Received 14 March 1996; accepted 17 April 1996**
